# Shades of Fine Dark Chocolate Colors: Polyphenol Metabolomics and Molecular Networking to Enlighten the Brown from the Black

**DOI:** 10.3390/metabo13050667

**Published:** 2023-05-17

**Authors:** Aecio Luís de Sousa Dias, Julie-Anne Fenger, Emmanuelle Meudec, Arnaud Verbaere, Pierre Costet, Clotilde Hue, Florent Coste, Sophie Lair, Véronique Cheynier, Jean-Claude Boulet, Nicolas Sommerer

**Affiliations:** 1 SPO, Université de Montpellier, INRAE, Institut Agro, F-34060 Montpellier, France; aecio-luis.de-sousa-dias@inrae.fr (A.L.d.S.D.); emmanuelle.meudec@inrae.fr (E.M.); arnaud.verbaere@inrae.fr (A.V.);; 2 INRAE, PROBE Research Infrastructure, PFP Polyphenol Analysis Facility, F-34060 Montpellier, France; 3 Valrhona, 26600 Tain-l’Hermitage, Franceflorent.coste@valrhona.fr (F.C.); sophie.lair@valrhona.fr (S.L.)

**Keywords:** *Theobroma cacao*, dark chocolates, metabolomics, feature-based molecular networking, phenolic compounds, procyanidins, flavanols, discriminating compounds

## Abstract

High-quality dark chocolates (70% cocoa content) can have shades from light to dark brown color. This work aimed at revealing compounds that discriminate black and brown chocolates. From 37 fine chocolate samples from years 2019 and 2020 provided by Valrhona,8 dark black samples and 8 light brown samples were selected. A non-targeted metabolomics study was performed based on ultra-high performance liquid chromatography—high resolution mass spectrometry/mass spectrometry experiments, univariate, multivariate, and feature-based molecular networking analyses. Twenty-seven overaccumulated discriminating compounds were found for black chocolates. Among them, glycosylated flavanols including monomers and glycosylated A-type procyanidin dimers and trimers were highly representative. Fifty overaccumulated discriminating compounds were found for brown chocolates. Most of them were B-type procyanidins (from trimers to nonamers). These phenolic compounds may be partially related to the chocolate colors as precursors of colored compounds. This study increases the knowledge on the chemical diversity of dark chocolates by providing new information about the phenolic profiles of black and brown chocolates.

## 1. Introduction

Chocolate is one of the most appreciated foods in the world. It is made from the seed kernels of the *Theobroma cacao* L. (Malvaceae) tree [1]. The cacao germplasm can be classified into 10 major genetic clusters [2]. The main cultivation areas of *T. cacao* are located in Africa and America. According to the International Cocoa Organization, these regions contributed 75% and 19%, respectively, of the total world production of 4,741,000 tons of cocoa beans in 2019–2020 [3]. Cocoa beans are initially processed by farmers through different stages including fermentation and drying. Additional steps, such as roasting, alkalization, and conching are carried out by manufacturers to obtain chocolate liquor, cocoa powder and butter which are ingredients of chocolate and other finished products [4]. The main categories of chocolate are dark, milk, and white, differing in their contents of cocoa solids, milk fat, and cocoa butter [1]. The chemical composition of chocolates depends on several variables related to the raw material, processing, and formulation. The composition of cocoa beans depends primarily on genetics, but also on geographical region of cultivation, agricultural practices, and climatic conditions [5].

The phenolic composition from cocoa beans to chocolate has been recently reviewed [5,6]. In general, unfermented cocoa beans have about 67 to 180 mg/g of total phenolic compounds, among which monomeric flavanols, proanthocyanidins, and anthocyanins correspond to approximately 37, 58, and 4%, respectively [5]. The phenolic composition is progressively modified and reduced during processing of cocoa beans. Fermentation can cause a reduction of up to 70% in phenolic compounds, mainly of monomeric flavanols (90%) and anthocyanins (93%) [6]. Drying can lead to a reduction between 26 and 77% of total polyphenols and up to 80% of flavanols [5]. Roasting is primarily responsible for the reduction of total phenolic compounds, mainly of monomeric flavanols (up to 95%) [5]. Consequently, the content of phenolic compounds in dark chocolates, which contain cocoa bean solids (up to 80% of the total weight), cocoa butter, and sugars [7], is very low in relation to the initial amount in unfermented cocoa beans. Martini and colleagues [8] reported a phenolic concentration of around 788 mg/100g for 70% dark chocolate. The main classes and proportions of phenolic compounds reported by these authors were flavanols (64%), including monomeric and oligomeric compounds, hydroxycinnamic acids (20.6%), ellagitannins (9.7%), hydroxybenzoic acids (3.1%), and flavonols (1.7%). Among other classes of compounds present in dark chocolates, peptides, alkaloids, and Maillard reaction products formed from reducing sugars and proteins as a consequence of processing at high temperatures can be mentioned [8,9,10]. Consumption of dark chocolate has been associated with health benefits. The most extensively studied biological effects of cocoa are the antioxidant, anti-inflammatory, cardiovascular, and metabolic effects which are mainly associated with phenolic compounds [7,9].

**Figure 1:**
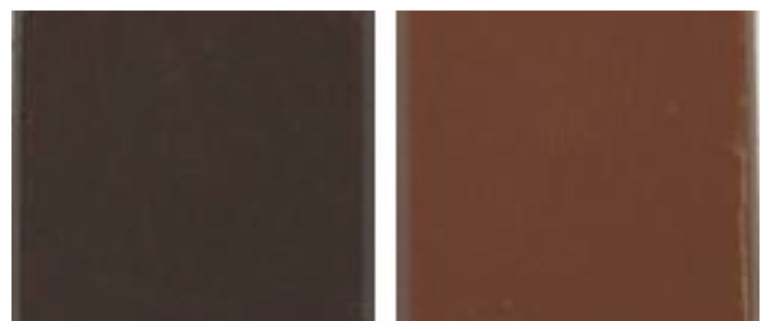
Example of dark chocolate (70% cocoa) with a black (**left**) and brown (**right**) color.

High-quality dark chocolates can have shades from light to dark brown color (Figure 1) [1]. To the best of our knowledge, the chemical composition of dark chocolates with a light brown color has not yet been studied. Color variations are related in part to the phenolic compounds, whose oxidation products contribute to the formation of colored compounds [11,12], and also to Maillard reaction products that correspond to brown polymeric compounds [5,13] that in turn may also associate with the phenolic compounds through covalent and non-covalent interactions [14]. Consequently, black and brown chocolates probably have discriminating phenolic compounds that could be markers for these products. 

Metabolomic approaches are able to detect a large number of molecules and to recognize compounds, discriminating different types of samples. They use successive steps including data acquisition, feature extraction, data processing, differential analysis, compound annotation, and identification [15]. Various statistical and visualization tools are used for differential analysis. Unsupervised tools such as principal components analysis (PCA) and hierarchical clustering, as well as supervised tools such as partial least squares-discriminant analysis (PLS-DA) [16,17] can be used to visualize patterns in datasets. In addition, statistical tests such as t-tests and univariate analysis of variance (ANOVA) can be performed. A common visualization for comparing two groups is the volcano plot that represents the fold change of signal abundances between the groups versus the *p*-value, the latter being a criterion to assess the statistical significance of signal differences between groups, in order to recognize the best discriminating compounds [15,16,17]. Then, the next challenge is the compound annotation. Different tools have been used such as feature-based molecular networking (FBMN) embedded within the Global Natural Products Social Molecular Networking (GNPS) infrastructure (https://gnps.ucsd.edu, accessed on 8 October 2022). Related molecules are connected according to their MS/MS spectra similarities, and results are presented as molecular networks. Moreover, the LC–MS feature abundances (peak areas) are used to estimate the relative ion abundance of each feature in each class [18,19]. Several mass spectrometry-based metabolomic studies on cocoa products have been performed to study metabolites, including phenolic compounds, related to the cultivar [20], geographical origin [20,21], fermentation process [22,23], and type of chocolate [18]. 

The aim of this work is to evaluate the chemical composition of dark chocolates of black and brown colors to try to reveal discriminating compounds of these types of chocolates. To achieve this goal, a non-targeted metabolomics study was performed using ultra-high performance liquid chromatography–high resolution mass spectrometry/mass spectrometry (UHPLC−HRMS/MS) experiments, univariate and multivariate statistical analysis, as well as FBMN analysis. This study is important not only to gain knowledge about the individual compounds but also to propose potential markers of these chocolates.

## 2. Materials and Methods

### 2.1. Materials

Standards of (+)-catechin, (−)-epicatechin, procyanidin dimer A2, protocatechuic acid, and 3-O-caffeoylquinic acid were purchased from Sigma-Aldrich (Steinheim, Germany), procyanidin B2 was purchased from Extrasynthese (Genay, France), procyanidin B3, and quercetin-3-O-glucoside were acquired from Phytolab (Vestenbergsgreuth, Germany), and procyanidin B5 was acquired from TransMIT PlantMetaChem (Giessen, Germany). LC−MS-grade acetonitrile and LC−MS-grade formic acid were from Biosolve (Valkenswaard, The Netherlands) and purified water was obtained from a Milli-Q water Millipore system (Bedford, MA, USA).

### 2.2. Chocolate Samples

Thirty-seven dark chocolates (70% cocoa), manufactured from commercial cocoa beans by the same process, were provided by Valrhona SA, Tain l’Hermitage, France. They were produced from beans harvested in the years 2019 and 2020 (Table 1). The industrial partner initially selected these samples based on a visual inspection of the chocolates in order to obtain samples of different shades of color. These chocolates were then classified more objectively as described in Section 2.3 and Section 2.4.

### 2.3. Color Analysis

The chocolate bars were melted in triplicate in an oven at 45 °C. The colors of melted chocolates were measured with a Chroma Meter CR-400 colorimeter from Konica Minolta (Sakay Osaka, Japan). The color parameters that describe the color perceived by the observer were expressed using the CIELAB system: Lightness (L* = 0 black to L* = 100 white), a* (a* < 0 green to a* > 0 red) and b* (b*< 0 blue to b* > 0 yellow). 

### 2.4. Color Classification Based on Visual Perception

The chocolate surfaces were not reproducible from one sample to another, yielding a variability in the light scattering which could alter the color perception in a sensory analysis of the chocolates bars. Thus, another more objective approach was undertaken. 

The L*, a*, and b* values were used to create an image of the color of each chocolate sample with Adobe Photoshop^®^ software [24]. A sensory analysis was performed on the color images of each chocolate. The images were transferred to a Microsoft PowerPoint slide and then distributed to 6 evaluators. All volunteer evaluators are employees of our research institute. They declared having no known deficiency that could influence color perception and had no difficulty recognizing colors in everyday life. The aim of the test was to classify the images into two groups, one containing the lightest brown chocolates and the other containing the darkest chocolates, so that each group was very homogeneous. These groups were called brown and black chocolates. The samples not classified into these two groups formed a third group called intermediate chocolates. The participants evaluated the images from a computer screen. They sorted the samples instinctively without much delay. The duration of the analysis was approximately thirty minutes. At the end of the test, the votes of the participants for each sample were recorded in a table. The samples that received the highest number of votes were definitively selected as brown or black chocolates, while the samples that received the fewest votes were classified as intermediate chocolates and eliminated from further analysis.

### 2.5. Extraction of Chocolate Samples

The extraction procedure on the black and brown chocolates was performed in triplicate based on previous works [25,26,27]. The chocolate was cut into small pieces and frozen with liquid nitrogen. The frozen sample was then ground in liquid nitrogen using a Pulverisette 2 analytical laboratory grinder from Fritsch (Idar-Oberstein, Germany). Approximately 360 mg of chocolate powder was transferred to test tubes and melted in an ultrasonic bath (40 °C). Lipids were extracted with 5 mL of hexane by sonication (40 °C, 30 min). The suspension was centrifuged (3000 rpm, 10 min). The supernatant was discarded and the pellet was dried using a Genevac vacuum evaporator at 35 °C (Ipswitch, UK).

The compounds of interest were extracted from the defatted residue. Fifteen milligrams of residue were extracted with 900 µL of acetone/water/acetic acid (70/28/2, *v*/*v*) in an ultrasonic bath (5 min, 28 °C). The mixture was centrifuged (15,000 rpm, 15 min). The precipitate was discarded and the supernatant was evaporated using the Genevac evaporator (35 °C). The dried material was suspended with 700 µL of methanol/water (80/20), sonicated (15 min), and centrifuged (15,000 rpm, 15 min). The supernatant was injected into the ultra-high performance liquid chromatography (UHPLC) system.

### 2.6. UHPLC−ESI−Q−Orbitrap MS Analyses

The black and brown chocolates were analyzed using a Vanquish UHPLC system from Thermo Fisher Scientific (Germering, Germany) that consisted of an autosampler VF-A10-A, a column compartment VH-C10-A, a diode array detector VF-D11-A, and a binary pump VF-P10-A. The column used was a Waters Acquity UPLC^®^ HSS T3 C18 column, 1.8 µm, 100 mm × 1.0 mm ID (Wexford, Ireland) equipped with an UltraShield UHPLC pre-column filter, 0.2 µm, from Restek Corporation (Lisses, France). Samples of 0.5 µL each were injected and the mobile phase was eluted with a constant flow rate of 220 µL/min, with the eluents being (A) 1% formic acid in water and (B) acetonitrile/water/ formic acid (80/19/1). The following gradient was used: 0–1.5 min: 2% B; 1.5–4.5 min: 2–12% B; 4.5–7 min: 12% B; 7–12 min: 12–24% B; 12–15 min: 24–48% B; 15–16 min: 48–60% B; 16–17 min: 60–100% B; 17–19 min: 100% B; 19–20 min: 100–2% B; 20–24 min: 2% B. Column and sample temperatures were maintained at 35 and 10 °C, respectively. 

The UHPLC system was hyphenated with an Orbitrap Exploris 480 mass spectrometer from Thermo Fisher Scientific (San José, CA, USA) equipped with an electrospray ionization (ESI) source operated in the negative ion mode using an internal post-source fluoranthen mass calibrant (radical ion at *m/z* 202.0788). The spray voltage was set to 2500 V. Sheath, auxiliary, and sweep gases were set to 35, 7. and 2 (arbitrary units), respectively. Ion transfer tube and vaporizer temperatures were set to 280 °C and 275 °C, respectively. The mass range used was from *m/z* 150 to *m/z* 1500. 

The injections occurred in the following order: a blank sample (solvent) was injected twice, followed by five consecutive injections of the quality control sample (mixture containing a fraction of each chocolate extract) at the beginning of a series of injections, then the chocolate extracts were randomly injected in triplicate, and after every eight injections of chocolate extracts, as well as at the end of the series of injections, a blank sample and a quality control sample were injected once, respectively.

These samples were injected in two series of analyses. The first (S1) was carried out to obtain HRMS spectra using the full scan mode and a resolution set to 240 k. A single quality control sample was injected with a resolution set to 480 k for identification purposes. The second series (S2) aimed to obtain HRMS and HRMS/MS spectra from two scan events. One event used the full scan mode with a resolution set to 60 k and the second event used the data-dependent acquisition mode to select the three most intense precursor ions from the first scan event to carry out the HRMS/MS experiments with a resolution set to 30 k. The precursor ions were fragmented in the HCD collision cell against nitrogen gas with the stepped normalized collision energy parameter set to 20–40–60%.

### 2.7. Chemometrics

The HRMS data from the S1 series were processed using 3.2 Compound Discoverer software. The values of the processing parameters of the UHPLC−HRMS data are described in the supplementary material (text document). The data processing resulted in a dataset where the rows corresponded to 371 features, each characterized by a retention time (RT) and *m/z* value. Each column presents the abundances of each feature of a sample.

First, unsupervised PCA was performed on the dataset using the 3.2 Compound Discoverer software, applying the center and scale options and normalized areas. Then, a volcano plot was used for feature selection. It compared two groups, brown versus black chocolates, for each feature. It was built as follows. The fold change was determined as the ratio of the brown/black mean areas, and a log_2_ transformation was applied to the fold change. Moreover, univariate ANOVA was performed on the same groups and the *p*-values were recorded. The lower the *p*-values, the higher the probability that the means of brown and black chocolates were different. A −log_10_ transformation was applied to the *p*-values. The volcano plot is the plot of the −log_10_ *p*-values versus the log_2_ fold change for each feature [15]. A threshold of −log_10_ *p*-value = 4 corresponding to a *p*-value of 0.0001 was used for the selection of the most discriminant features.

Then, the second goal was to annotate the discriminating features. FBMN analysis was performed to help with the annotation process, based on the correlations between the MS/MS spectra of the samples visualized as molecular networks. Moreover, the LC–MS feature abundances were used to estimate the relative ion abundance of each feature in each class of samples. The FBMN analysis was carried out as follows. The MS and MS/MS data in raw format obtained from the S2 analysis series were converted to .mzML format, using the MSConvert tool (version 3). Data were then processed with MZmine 3 [28] using processing parameters whose values are described in the supplementary material (text document). The results of this processing consisted of the feature quantification table (.CSV file format) containing the abundances of 562 LC−MS features, and the MS/MS spectral summary (.MGF file) with a representative MS/MS spectrum per LC-MS feature. These files were exported to GNPS [29] for FBMN analysis from the FBMN workflow [19]. The precursor ion mass tolerance was set to 0.02 Da and the MS/MS fragment ion tolerance to 0.02 Da. The molecular networks were then created where edges were filtered to have a cosine score above 0.7 and more than 6 matched peaks. The molecular networks were visualized using Cytoscape software (version 3.9.1) [30]. Manual annotation of features was performed based on predicted molecular formulae, fragmentation patterns, and comparison to literature.

## 3. Results and Discussion

### 3.1. Sample Classification from Visual Evaluation

Color analysis was performed on the 37 chocolates. Table S1 shows the L*, a*, and b* values of the samples arranged in descending order of a* parameter. The color image of each chocolate was reconstituted from the values of L*, a*, and b* parameters for sensory analysis. 

**Figure 2:**
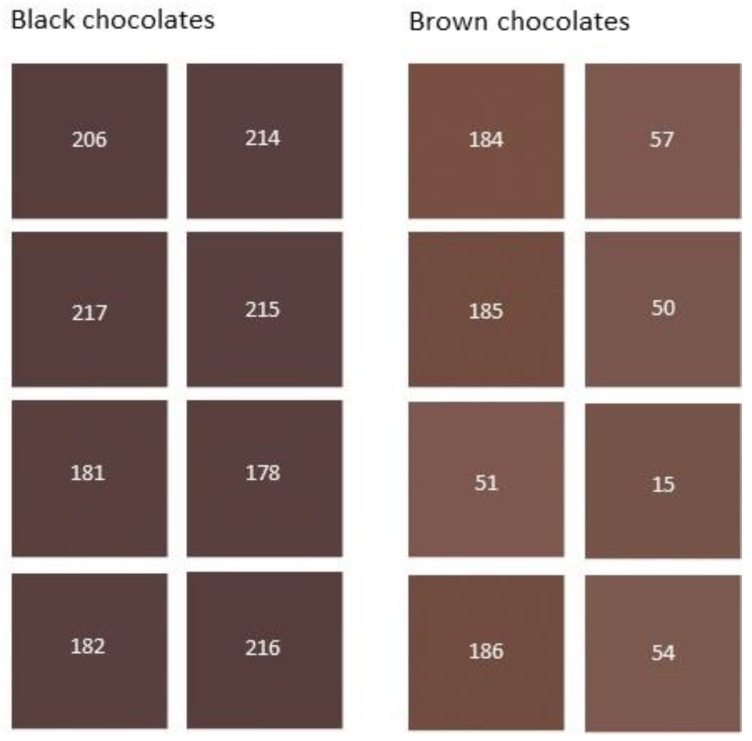
Color images of black and brown chocolate groups obtained from their L*, a* b* color parameters by Adobe PhotoShop^®^. The numbers correspond to the code samples in Table 1.

All participants in the sensory analysis classified eight samples (codes 184, 185, 186, 15, 50, 51, 54, and 57) as brown chocolates (Table S2). Therefore, these samples were selected to form the group of brown chocolates. Similarly, eight samples (codes 216, 214, 217, 206, 178, 215, 181, and 182) were retained to build the black chocolate group. Indeed, these samples received the votes of all participants, except for sample 182 which received 5 out of 6 votes (Table S2). The fact that the selected chocolates received the votes of at least 5 evaluators demonstrates confidence in the brown and black chocolate groups that were obtained. The rest of the chocolate samples were classified having an intermediate color and were eliminated from the further stages of the study. Figure 2 shows the reconstituted colors of the black and brown chocolate groups selected by the evaluators.

The ranges of L*, a*, and b* values of the black chocolates were 29.3–29.9, 10.8–11.3, and 4.8–6.0, respectively (Table S1). Lower values of L* and a* have been previously reported in the literature for dark chocolates (about 25, 7, and 7 for L*, a*, and b* values, respectively) [31,32]. In general, the black chocolates had lower values of L*, a*, and b* than brown chocolates whose ranges were 35.7–41.1, 14.3–15.6, and 11.2–15.9, for L*, a*, and b* values, respectively.

The ranges of L*, a*, and b* values found by Briones and co-workers [33] for milk chocolates were L* = 35–45, a* = 6–8, and b* = 7–10. The L* values were similar, but brown chocolates showed higher a* and b* values. Ramos-Escudero and colleagues [34] also describe higher values of L*, a*, and b* (L* = 33.74, a* = 14.78, and b* = 8.47) for cocoa kernels with lighter brown color compared to the darker cocoa kernels (L* = 16.82–17.51, a* = 4.11–5.22, and b* = 2.31–3.29). The high values of a* and b* observed for brown chocolates in the present study may be related to yellow, brown, and red colored compounds resulting from transformation of phenolic compounds [11]. This reference shows that, among the cocoa beans roasted under variable air-flow rate, the sample that showed the highest amounts of these colored compounds also showed the highest values of a* and b*, which were also accompanied by a higher value of L* (L* = 34.68, a* = 12.49, and b* = 7.42). In that same study, the sample that presented the deepest dark brown color had the lowest value of L* (L* = 31.10, a* = 8.09, and b* = 5.33) [11].

In general, the intermediate chocolates showed intermediate values of the L*, a*, and b* parameters in comparison with brown and black chocolates. Their L*, a*, and b* ranges were 30.7–38.4, 11–14.5, and 4.5–11.3, respectively. 

### 3.2. Data Processing and Statistical Analysis

The 16 selected black and brown chocolate samples (Table S2) were then analyzed by UHPLC−ESI−Q−Orbitrap MS. Features from the UHPLC−HRMS data were extracted by the 3.2 Compound Discoverer software, resulting in a dataset of 371 features. Table S3 shows the values of RT and measured *m/z* of the selected features as well as their chromatographic peak areas for each sample.

**Figure 3:**
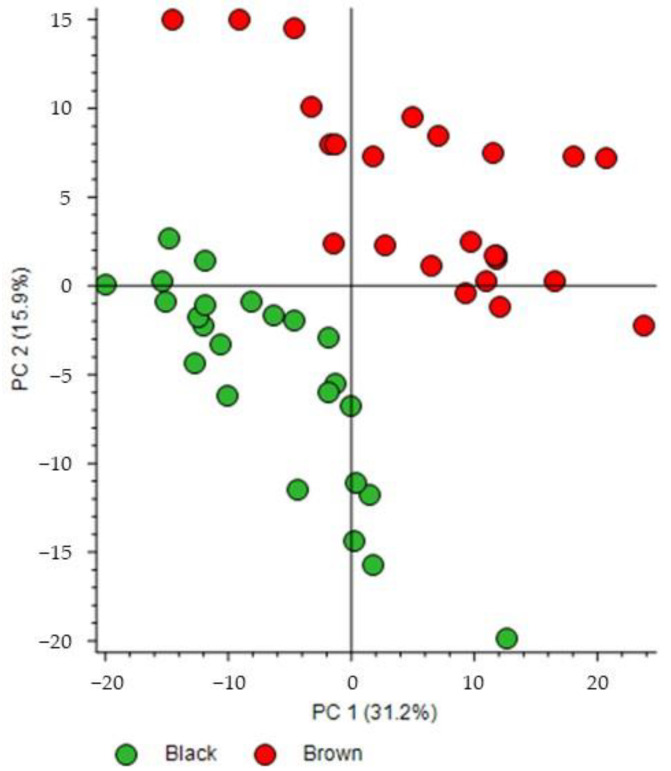
Score plot of principal component analysis from the UHPLC−HRMS features of the black and brown chocolates.

**Figure 4:**
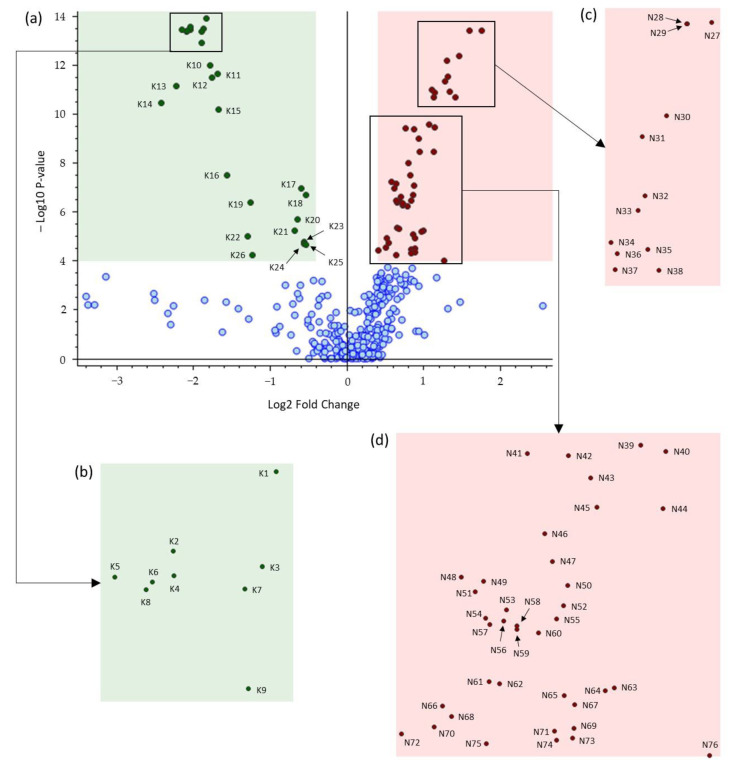
(**a**) Volcano plot comparing brown versus black chocolates. Red and green dots represent overaccumulated discriminating compounds for brown and black chocolates, respectively, and blue dots indicate metabolites that were not highly discriminating for brown and black chocolates (*p*-value: < 0.0001; Log2 Fold Change: 0.4). (**b**–**d**) are zoomed regions of (**a**). The feature codes correspond to the compound codes listed in Table S4.

The score plot of the PCA performed on the dataset shows good separation between the brown and black chocolate groups (Figure 3). Principal components 1 and 2 (PC1 and PC2) accounted for 31.2% and 15.9% of the variability, respectively. Both PC1 and PC2 contributed to this separation. In general, black chocolates were characterized by low scores on both PC1 and PC2 whereas brown chocolates were characterized by higher PC1 and PC2 scores for most samples. Then, the same dataset (Table S3) was used to obtain the volcano plot (Figure 4a) for selection of the most discriminating features in the two groups. According to the fold change calculation described in the Experiment section, overaccumulated compounds of brown and black chocolates were located on the right and left hand sides of the graph, respectively. For example, for a value of +3 on the abscissa axis of a given feature, the mean area of brown chocolates is 2³ = 8 times higher than that of black chocolates; and for a value of −3, the mean area of brown chocolates is 2^−3^ = 1/8 of that of black chocolates. Although no regulation mechanism is presented in our study, by analogy with terms introduced in the literature [15,16,17], features on the right hand side would be upregulated for brown chocolates but downregulated for black chocolates and features on the left hand side would be upregulated for black but downregulated for brown chocolates.

Features above and under the threshold of −log_10_ *p*-value = 4 on the ordinate axis corresponding to a *p*-value of 0.0001 were qualified as highly and non-highly discriminant features, respectively (Figure 4a). Therefore, the pink shaded region on the right of Figure 4a contains the most discriminating overaccumulated features for brown chocolates (50 metabolites coded from N27 to N76) and the green shaded region on the left contains the most discriminating overaccumulated features for black chocolates (26 metabolites coded as K1–K26). 

These discriminating features are highlighted in Figure S1, corresponding to the loading plot of the PCA described earlier. This loading plot also indicates that high levels of these discriminating features contributed to the separation between black and brown chocolates observed in the score plot of the PCA in Figure 3. 

These features may be potential markers of brown and dark chocolates. Some works have used metabolomic approaches including the use of volcano plots to study chemical markers related to colors in different matrices, such as rice [16] and potatoes [17]. To the best of our knowledge, this is the first study on chemical markers of brown and black colors in dark chocolates.

### 3.3. Compound Identification

#### 3.3.1. Identification of Discriminating Compounds for Black Chocolates

The most discriminating features selected in the previous section (Figure 4) for the black and brown chocolates were tentatively annotated based on their UHPLC−HRMS/MS information described in Table S4 and comparison with the literature. They are listed in Table S4 in descending order of significance, according to Figure 4. The MS/MS fragmentation data of compounds that have already been identified in other works cited in Table S4 are not discussed here, except when necessary.

The K1 feature was identified as protocatechuic acid, by comparison with an authentic standard (Table S4). It was the only phenolic acid among the discriminating compounds. Protocatechuic acid is also present in cocoa beans [35] and is the major hydroxybenzoic acid reported in other studies on dark chocolates [8,18]. 

**Figure 5:**
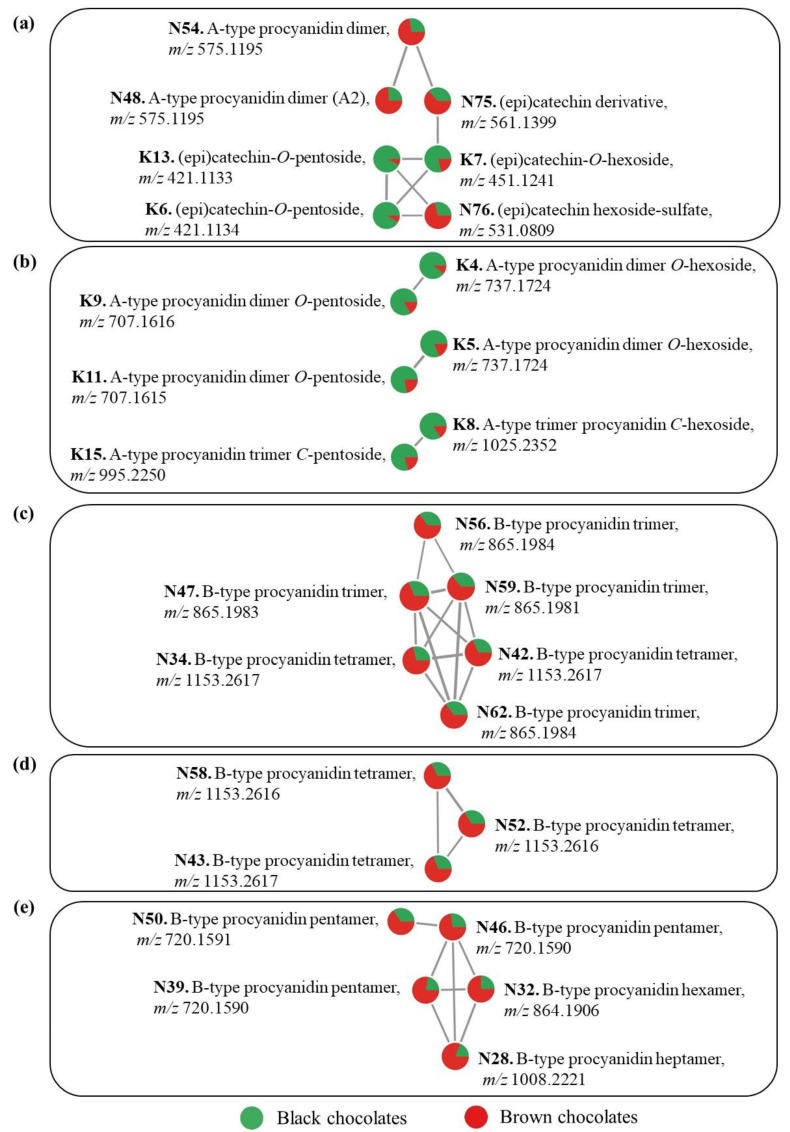
Feature-based molecular networks (**a**–**e**) of discriminating compounds of black and brown chocolates. Full analytical details of the compounds are available in Table S4. Pie charts represent the relative abundance of each compound in the black and brown chocolates.

Six *O*-glycosylated flavan-3-ol monomers were tentatively annotated (K3, K6, K7, K10, K12, and K13) (Table S4). Features K3 and K7 detected at *m/z* 451.124 (for values with four decimals, see Table S4) corresponded to (epi)catechin-*O*-hexosides, because their MS/MS fragmentation showed fragments at *m/z* 289.072 corresponding to an aglycone (epi)catechin and a neutral loss of 162 u, which is consistent with an *O*-hexoside moiety. Similarly, features K10 and K12 detected at *m/z* 467.119 corresponded to (epi)gallocatechin-*O*-hexoside, based on their fragments at *m/z* 305.066 and *m/z* 305.067, respectively, that indicated an aglycone (epi)gallocatechin and a neutral loss of an *O*-hexoside. All of these compounds have previously been detected in dark chocolates [8]. The FBMN analysis showed that features K6 and K13 (*m/z* 421.113) are connected with feature K7 in a molecular network (Figure 5a), reflected in these compounds’ MS/MS spectral similarity. The most abundant fragments of features K6 and K13 at *m/z* 289.072 correspond to (epi)catechin aglycone resulting from loss of a pentose (132 u) (Table S4) [36]. Therefore, K6 et K13 were both tentatively identified as (epi)catechin-*O*-pentosides. This may be the first report of these compounds in dark chocolates, although an (epi)catechin-*C*-pentoside was previously reported [8]. The FBMN analysis also shows that features K6, K7, and K13 in Figure 5a were more abundant in black than in brown chocolates which is in agreement with what was observed in the volcano plot (Figure 3). Martini and colleagues [8] observed that among the glycosylated monomeric flavanols of a dark chocolate, an (epi)catechin-*O*-hexoside was more abundant than the other phenolic compounds.

Features K9, K11 and K14 (*m/z* 707.162) were tentatively annotated as procyanidin A-type dimer *O*-pentosides. Their MS/MS fragments at *m/z* 581.130 and *m/z* 449.087 or *m/z* 499.088, resulting from a sequential loss of 126 u (a heterocyclic ring fission) and 132 u (loss of a pentose), suggest the presence of an *O*-pentoside moiety (Table S4). Both features K4 and K5 (*m/z* 737.172) presented fragments at *m/z* 611.140 (or *m/z* 611.141) and *m/z* 449.087, corresponding to a sequential loss of 126 u and 162 u (loss of a hexose) (Table S4), suggesting procyanidin A-type dimer *O*-hexosides. Additionally, the molecular networks in Figure 5b show the molecular correspondence between the feature pairs K9 and K4, and K11 and K5, and the higher proportion of these compounds in black compared with brown chocolates. All these compounds have previously been detected in dark chocolates [8,18].

Features K8 and K15 (*m/z* 1025.235 and *m/z* 995.225) were tentatively annotated as A-type procyanidin trimer hexoside and A-type procyanidin trimer pentoside, respectively, based on their HRMS/MS data (Table S4). These compounds were found earlier by D’Souza and co-workers [23] in unfermented and fermented cocoa beans, and a A-type procyanidin trimer hexoside has previously been detected in dark chocolates [8]. However, it was not possible to determine whether K8 and K15 are *O*- or *C*-glycosides based on the obtained fragments. They were also connected in a molecular network (Figure 5b).

The HRMS/MS data of feature K2 (*m/z* 573.1038) corresponded to an oxidized A-type procyanidin dimer (Table S4) as found after degradation of procyanidin A2 in a model medium [37]. However, the position of the third interunit linkage in K2 could not be determined. A-type procyanidins can be formed from B-type procyanidins by oxidation processes [38]. They also can undergo further oxidation to generate other products such as oxidized A-type procyanidins [37]. As far as we know, this is the first time that this type of compound has been evidenced in chocolate, but it has previously been observed in a cider apple juice [39].

Feature K23 (*m/z* 210.0772) was characterized as the amino acid derivative methoxytyrosine, as also identified by Vargas-Arana et al. in cocoa waste flours [40]. Finally, a few discriminating compounds were not identified, such as K16–K22, and K24–K26, but their HRMS/MS spectral data are described in Table S4 as a basis for future studies.

#### 3.3.2. Identification of Discriminating Compounds for Brown Chocolates

Feature N44 (*m/z* 359.0977) corresponds to a syringic acid hexoside previously identified in dark chocolates [8]. Feature N75 at *m/z* 561.1399 had fragments at *m/z* 289.0716, *m/z* 245.0820, and *m/z* 125.0244 which indicate that this compound is an (epi)catechin derivative [41]. Similarly, feature N76 at *m/z* 531.0809 had MS/MS fragments at *m/z* 289.0717, *m/z* 245.0820, *m/z* 137.0245, and *m/z* 125.0242. Both N75 and N76 features may be glycosylated, as supported by their connection to glycosylated (epi)catechins (K6, K13, and K7) in a molecular network (Figure 5a). The figure also shows the highest relative abundance of these compounds in brown chocolates. Additionally, regarding feature N76, the characteristic M+2 intra-isotopic profile obtained at high resolution (R = 480 k, data not shown) indicated the unambiguous presence of a sulfur atom in the molecule. A sulfate group is probably linked to a hexoside because the MS/MS fragmentation of the molecular ion directly generated the fragments related to the (epi)catechin aglycone. Therefore, this feature was identified as an (epi)catechin hexoside-sulfate. This may be the first report of this type of compound in dark chocolate.

The HRMS/MS fragmentation data of features N45 and N53 (*m/z* 577.1350) corresponded to dehydrodi(epi)catechins B with a *β*-interflavanic configuration, as reported by Dias and colleagues [42]. These compounds are formed by autoxidation or enzymatic oxidation by polyphenoloxidase (PPO), laccase, tyrosinase, and peroxidase from flavan-3-ols such as (+)-catechin and (−)-epicatechin [42].

Two A-type procyanidin dimers were identified (N48 and N54, *m/z* 575.1195), as in previous works on dark chocolates [8,18]. These major compounds in brown chocolates are represented in Figure 5a. As discussed for black chocolates, A-type procyanidins may be formed from B-type procyanidins through oxidation processes [37]. Epimers of procyanidin A-type dimers have been reported as markers of fermented cocoa beans [10]. Feature N71 (*m/z* 739.1881) was annotated as a procyanidin B-type dimer *C*-hexoside which was previously detected in dark chocolates [8]. Its MS/MS fragments at *m/z* 649.1564 and *m/z* 619.1460 corresponding to the losses of 90 u and 120 u, respectively, are characteristic of *C*-hexoside compounds [36]. Flavan-3-ol-*C*-glycosylated compounds can be synthesized through enzymatic and also non-enzymatic reactions [43]. In the latter case, flavan-3-ols were *C*-glycosylated in the C6 or C8 positions after their condensation reactions with the open-chain form of the glucose after alkalization on the cocoa powder [44]. Similarly, ethyl bridged flavan-3-ols and epicatechin-ethyl-procyanidins were found in fermented cocoa beans as a result of the condensation of flavan-3-ols with acetaldehyde in the C6 and C8 positions of the flavan-3-ol units [45]. Perhaps non-enzymatic *C*-glycosylation also occurs with procyanidins and, if this is the case, the N71 compound may have been produced during the chocolate manufacturing process.

Eight B-type procyanidin trimers (N47, N49, N56, N59, N61, N62, N65, and N55) and 7 B-type procyanidin tetramers (N34, N37, N42, N43, N52, N58, and N60) that are known compounds in dark chocolates [8,25] were tentatively identified based on their HRMS/MS data [8,18,23,46] (Table S4). Feature N55 (*m/z* 1027.2509) had a MS/MS fragment at *m/z* 907.2056 corresponding to a loss of 120 u, which suggests the presence of a *C*-hexose in its structure, as with feature N71, previously discussed [36]. These groups of oligomers, abundant in brown chocolates, were assembled into two molecular networks (Figure 5c,d).

Similarly, pentameric (N46, N31, N36, N39, N40, and N50), hexameric (N30, N32, N33, and N38), and heptameric (N28) B-type procyanidins were tentatively identified. Their HRMS/MS spectral data are in accordance with the literature for chocolate [8,18,25]. Furthermore, some of these compounds (N39, N50, N46, N32, and N28) were connected in the same molecular network (Figure 5e), which facilitated their chemical annotations. The figure also shows the greater relative abundance of these compounds in brown chocolates.

The HRMS information and MS/MS fragmentation pattern data allowed tentative identification of octameric (N29) and nonameric (N27) B-type procyanidins [23,47]. Oligomeric procyanidins, up to decamers, have been reported in cocoa and chocolate [5]. 

Two amino acid derivatives were tentatively identified as feruloyl aspartic acid (N63) [8,20] and phenylalanylphenylalanine (N72) [48] according to their MS/MS fragmentation patterns. The first of these and amino acids such as phenylalanine, tryptophan, and tyrosine are common compounds found in chocolates [8,18].

A minority of the discriminating compounds (N35, N41, N51, N57, N64, N66-N70, N73, and N74) could not be identified based on their chromatographic and MS spectral data (Table S4).

Finally, the different phenolic profiles of brown and black chocolates suggest that these chocolates may have different impacts on human health, but this needs to be proven in further studies. In terms of catechin derivatives, monomers have a higher bioavailability than oligomers and polymers. On the other hand, oligomers and polymers can be metabolized by the gut microbiota into metabolites that can be absorbed and have a systemic effect. In addition, polymers can also exert a local effect in the intestinal tract [49]. 

### 3.4. Considerations on the Origin of Colors

The chemical composition of chocolates depends on many factors, including the initial cocoa bean composition, genetics, geographical region of cultivation, agricultural practices, climatic factors, and also the cocoa processing, that are also responsible for the formation of the characteristic colors of chocolates [5]. 

As described in the Introduction section, the main phenolic compounds and their proportions in unfermented cocoa beans are monomeric flavanols (37%), proanthocyanidins (58%), and anthocyanins (4%) [5]. These phenolics are progressively modified and drastically reduced during processing of cocoa beans. The amount of anthocyanins is almost entirely reduced during fermentation [6]. Similarly, the monomeric flavanols are radically reduced during fermentation, drying, and roasting [5,6] and polymerized to procyanidins during roasting [6]. Consequently, the content of phenolic compounds in dark chocolates is very low in relation to the initial amount in unfermented cocoa beans and is the result of several chemical transformations. 

Concerning the studied discriminating compounds, *O*-glycosylated flavan-3-ol monomers (K3, K6, K7, K10, K12, and K13) may be related to the color of black chocolates, in which they were overaccumulated, but also of brown chocolates where they were present in lower proportions (Figure 4) as explained in the Data Processing and Statistical Analysis section. Indeed, flavan-3-ols are precursors of colored compounds formed after enzymatic and non-enzymatic oxidation. Yellow and brown compounds are formed on cocoa beans during fermentation and drying processes [50], such as dehydrodi(epi)catechins A formed from the colorless intermediate product dehydrodi(epi)catechins B [51], that were also found in the present work (compounds N45 and N53). Additionally, dehydro(epi)catechin oligomers may have a degree of polymerization of 2 up to at least 15 (epi)catechin subunits, as described by Verloop and co-workers for black tea [52]. Germann and colleagues [53] identified yellow (dehydrocatechinic acid dimers) and red chromophores (hydroxycatechinic acids and their radical states) formed from flavan-3-ols after cocoa powder alkalization. These authors also observed that hydroxycatechinic acids polymerized to reddish-brown chromophores with molecular weight >10 kDa that were the major contributors of cocoa darkening. In another work, Germann and colleagues [54] also identified reddish-colored xanthenocatechins derived from flavan-3-ols in alkalized cocoa powder. 

Probably, oxidation products of procyanidins formed during processing also contributed to the color mainly in brown chocolates where a high number of procyanidins were overaccumulated. Procyanidin oxidation products were the direct contributors to the brown color of cotton fibers [55].

Compounds derived from amino acids such as N72 and K23 may contribute to chocolate colors, by their participation in Maillard reactions that can occur during drying, roasting, and conching [5]. Moreover, several bound phenolic compounds contained in high-molecular weight melanoidin fractions (brown Maillard reaction products) from roasted cocoa beans were identified after acidic and alkaline hydrolysis [14]. Some of the bound phenolics described in this work were protocatechuic acid, (+)-catechin, (−)-epicatechin, procyanidin B2, and caffeoyl aspartic acid. Some of these compounds or similar compounds were also overaccumulated in brown or black chocolates in the present work, such as some phenolic acids (K1 and N44), several flavan-3-ol monomers (K3, K6, K7, K10, K12, and K13), several procyanidin dimers (K4, K5, K9, K11, K14, N48, N54, and N71) and trimers (K8, K15, N47, N49, N56, N59, N61, N62, N65, and N55), and feruloyl aspartic acid (N63). These compounds may have also contributed to the colors of chocolates as bound phenolics in melanoidins.

## 4. Conclusions

This work aimed to study the composition of high-quality dark chocolates (70% cocoa content) with black and light brown color, in order to determine discriminating compounds of these two types of chocolates. The metabolomic study included non-targeted UHPLC−HRMS/MS experiments, univariate and multivariate analyses, as well as feature-based molecular networking analysis, identifying 27 discriminating compounds for black chocolates, of which 16 were tentatively annotated, and 50 discriminating compounds for brown chocolates (38 annotated). 

For the black chocolates, the compounds were mainly glycosylated A-type procyanidin dimers and trimers (seven compounds) and *O*-glycosylated flavan-3-ol monomers (six compounds), but also one oxidized A-type procyanidin dimer, one phenolic acid, and one amino acid derivative. Regarding the brown chocolates, the discriminating compounds corresponded mainly to non-glycosylated B-type procyanidins with degrees of polymerization between 3 and 9 (twenty-seven compounds), along with *C*-glycosylated B-type procyanidins (one dimer and one trimer), two A-type procyanidin dimers, two dehydrodicatechins B, one phenolic acid, and two amino acid derivatives. The discriminating compounds found in this work can potentially be markers of these types of chocolates.

These compounds may be indirectly related to chocolate colors as precursors of yellow, brown, and red colored compounds formed after oxidation or as bound phenolics contained in brown Maillard reaction products. Differences in phenolic and color profiles between black and brown chocolates may be related to genetic and pedological factors, and fermentation and drying conditions. 

The findings of this work need to be confirmed by other studies with a larger number of samples. Future metabolomic studies may also be carried out on the raw materials and on intermediate-colored chocolates.

## Figures and Tables

**Table 1:** Description of chocolate samples from Brazil.

Color Classification ^a^	Sample Code (Year ^b^)
Brown	184 (2019), 185 (2019), 51 (2020), 186 (2019), 57 (2020), 50 (2020), 15 (2020), 54 (2020)
Intermediate	183 (2019), 17 (2020), 19 (2020), 55 (2020), 20 (2020), 56 (2020), 18 (2020), 14 (2020), 58 (2020), 180 (2019), 13 (2020), 16 (2020), 21 (2020), 53 (2020), 205 (2019), 210 (2019), 52 (2020), 204 (2019), 179 (2019), 209 (2019), 207 (2019)
Black	206 (2019), 217 (2019), 182 (2019), 214 (2019), 215 (2019), 181 (2019), 178 (2019), 216 (2019)

^a^ The samples were classified according to the images of their colors formed from the L*, a*, and b* values (Table S1) that were evaluated in a sensory analysis (Table S2). ^b^ The samples were produced from beans harvested in the years 2019 or 2020.

## Data Availability

The data presented in this study are available on request from the corresponding author. The data are not publicly available due to being commercial property.

## Supplementary Materials

The following supporting information can be downloaded at: https://www.mdpi.com/article/10.3390/metabo13050667/s1, Figure S1: Loading plot of principal component analysis from the UHPLC−HRMS features of the black and brown chocolates; Table S1: L*, a*, and b* color parameters for chocolate samples; Table S2: Sensory analysis of the reconstituted images of the colors of the chocolates; Table S3: dataset; Table S4: Highly discriminating compounds for black and brown chocolates tentatively annotated by UHPLC−ESI−Q−Orbitrap MS analyses.
