# Supplementary material for: Shades of Fine Dark Chocolate Colors: Polyphenol Metabolomics and Molecular Networking to Enlighten the Brown from the Black
Source: Metabolites. 2023 May 17;13(5):667. doi: 10.3390/metabo13050667 (PMC10224107; doi:10.3390/metabo13050667)

Supplementary material

# Shades of Fine Dark Chocolate Colors: Polyphenol Metabolomics and Molecular Networking to Enlighten the Brown from the Black

**Aecio Luís de Sousa Dias [1,2], Julie-Anne Fenger [1,2], Emmanuelle Meudec [1,2], Arnaud Verbaere [1,2], Pierre Costet [3], Clotilde Hue [3], Florent Coste [3], Sophie Lair [3], Véronique Cheynier [1,2], Jean-Claude Boulet [1,2,*] and Nicolas Sommerer [1,2,*]**

[1] SPO, Université de Montpellier, INRAE, Institut Agro, F-34060 Montpellier, France; aecio-luis.de-sousa-dias@inrae.fr (A.L.d.S.D.); emmanuelle.meudec@inrae.fr (E.M.); arnaud.verbaere@inrae.fr (A.V.)
[2] INRAE, PROBE Research Infrastructure, PFP Polyphenol Analysis Facility, F-34060 Montpellier, France
[3] Valrhona, 26600 Tain-l'Hermitage, France; florent.coste@valrhona.fr (F.C.); sophie.lair@valrhona.fr (S.L.)
* Correspondence: jean-claude.boulet@inrae.fr (J.-C.B.); nicolas.sommerer@inrae.fr (N.S.)

## Description of the Compound Discoverer software processing parameters used for raw UHPLC−HRMS data processing and selection of features

*Select Spectra*
Spectrum Properties Filter:
- Lower RT Limit:  0.3
- Upper RT Limit:  17
- Lowest Charge State:  0
- Highest Charge State:  3
- Min. Precursor Mass:  130 Da
- Max. Precursor Mass:  1800 Da
- Total Intensity Threshold:  0
- Minimum Peak Count:  1

Peak Filters:
- S/N Threshold (FT-only):  3

General Settings:
- Precursor Selection:  Use MS(n - 1) Precursor
- Use Isotope Pattern in Precursor Reevaluation:  True

*Align Retention Times*
General Settings:
- Alignment Model:  Adaptive curve
- Alignment Fallback:  None
- Maximum Shift [min]:  0.5
- Mass Tolerance:  1 ppm
- Remove Outlier:  True

*Detect Compounds*
General Settings:
- Mass Tolerance [ppm]:  1 ppm
- Intensity Tolerance [%]:  50
- S/N Threshold:  2
- Min. Peak Intensity:  500000

- Ions: [2M-H]-1; [M+Cl]-1; [M+Formic Acid-H]-1; [M-2H]-2; [M-H]-1
- Base Ions: [M-H]-1
- Min. Element Counts: C H O
- Max. Element Counts: C190 H190 N8 O120 S2

Peak Detection:
- Filter Peaks: True
- Max. Peak Width [min]: 0.5
- Remove Singlets: True
- Min. # Scans per Peak: 5
- Min. # Isotopes: 2

Isotope Grouping:
- Min. Spectral Distance Score: 0
- Remove Potentially False Positive Isotopes: True

*Group Compounds*
Compound Consolidation:
- Mass Tolerance: 2 ppm
- RT Tolerance [min]: 0.5

*Fill Gaps*
General Settings:
- Mass Tolerance: 2 ppm
- S/N Threshold: 2

*Apply Missing Value Imputation*
General Settings:
- Imputation Method: Median + Small Value with Variability

*Apply QC Correction*
General Settings:
- Regression Model: Linear
- Min. QC Coverage [%]: 50
- Max. QC Area RSD [%]: 30
- Max. Corrected QC Area RSD [%]: 25
- Max. # Files Between QC Files: 15

*Mark Background Compounds*
General Settings:
- Max. Sample/Blank: 5
- Max. Blank/Sample: 0
- Hide Background: True

**Description of the MZmine software processing parameters used for raw UHPLC−HRMS/MS data and feature selection before feature-based molecular networking analysis**

*Mass detection*
Scans: MS level (1)
Scan types: All scan types
Mass detector: Centroid
Noise level: 100000.0
Detect isotope signals below noise level: false
m/z tolerance: 5.0E-4 m/z or 10.0 ppm
Maximum charge of isotope m/z: 1

Scans: MS level (2)
Scan types: All scan types
Mass detector: Centroid
Noise level: 1000.0
Detect isotope signals below noise level: false
m/z tolerance: 5.0E-4 m/z or 10.0 ppm
Maximum charge of isotope m/z: 1

*ADAP Chromatogram Builder*
Scans: MS level (1)
Group intensity threshold: 300000.0
Min highest intensity: 300000.0
Scan to scan accuracy (m/z): 0.0 m/z or 10.0 ppm

*Local minimum feature resolver*
MS/MS scan pairing: true
Retention time tolerance: 0.2 minutes
MS1 to MS2 precursor tolerance (m/z): 0.01 m/z or 10.0 ppm
Dimension: Retention time
Chromatographic threshold: 0.9
Minimum relative height: 0.1
Minimum absolute height: 300000.0
Min ratio of peak top/edge: 2.0
Peak duration range (min/mobility): [0.02..2.0]
Min # of data points: 3

*13C isotope filter (formerly: isotope grouper)*
m/z tolerance: 0.0 m/z or 10.0 ppm
Retention time tolerance: 0.03 minutes
Maximum charge: 2
Representative isotope: Most intense
Never remove feature with MS2: true

*Join aligner*
m/z tolerance: 0.0 m/z or 10.0 ppm
Weight for m/z: 75.0
Retention time tolerance: 0.05 minutes
Weight for RT: 25.0
Require same charge state: true

*Filtering feature list rows*

Minimum features in a row (abs or %): true (2.0)
Minimum features in an isotope pattern: true (2)
Validate 13C isotope pattern: true
    m/z tolerance: 5.0E-4 m/z or 10.0 ppm
    Max charge: 2
    Estimate minimum carbon: true
    Remove if 13C: true
Feature with MS2 scan: true
Never remove feature with MS2: true

*Feature list blank subtraction*
Minimum # of detection in blanks: 2
Fold change increase: true (4.0)

*Gap filling*
Intensity tolerance: 0.1
m/z tolerance: 0.0 m/z or 10.0 ppm
Retention time tolerance: 0.05 minutes
Minimum data points: 1

*Duplicate feature list rows filter*
Filter mode: NEW AVERAGE
m/z tolerance: 0.0 m/z or 5.0 ppm
RT tolerance: 0.07 minutes

**Table S1** : L*, a* and b* color parameters for chocolate samples

| Sample code [a] | Color classification [b] | L* [c] | a* [c] | b* [c] |
|---|---|---|---|---|
| 184 | N | 38.3 ± 0.13 | 15.6 ± 0.05 | 15.9 ± 0.10 |
| 185 | N | 35.7 ± 0.16 | 15.1 ± 0.09 | 13.7 ± 0.12 |
| 51 | N | 41.1 ± 0.16 | 14.8 ± 0.14 | 12.4 ± 0.09 |
| 186 | N | 35.7 ± 0.21 | 14.6 ± 0.06 | 13.6 ± 0.15 |
| 57 | N | 41.0 ± 0.30 | 14.6 ± 0.10 | 12.1 ± 0.12 |
| 50 | N | 40.4 ± 0.26 | 14.5 ± 0.24 | 11.2 ± 0.17 |
| 183 | I | 34.1 ± 0.12 | 14.5 ± 0.08 | 11.3 ± 0.12 |
| 15 | N | 39.3 ± 0.19 | 14.3 ± 0.01 | 11.7 ± 0.13 |
| 54 | N | 40.8 ± 0.13 | 14.3 ± 0.33 | 11.6 ± 0.49 |
| 17 | I | 38.4 ± 0.19 | 14.1 ± 0.09 | 8.9 ± 0.08 |
| 19 | I | 37.7 ± 0.15 | 14.1 ± 0.20 | 8.2 ± 0.23 |
| 55 | I | 37.8 ± 0.27 | 14.0 ± 0.06 | 7.0 ± 0.14 |
| 20 | I | 37.7 ± 0.08 | 13.5 ± 0.06 | 8.3 ± 0.03 |
| 56 | I | 38.4 ± 0.27 | 13.4 ± 0.19 | 8.7 ± 0.07 |
| 18 | I | 37.1 ± 0.25 | 13.3 ± 0.07 | 7.5 ± 0.04 |
| 14 | I | 37.2 ± 0.20 | 13.2 ± 0.13 | 7.5 ± 0.01 |
| 58 | I | 36.2 ± 0.39 | 12.8 ± 0.11 | 6.5 ± 0.03 |
| 180 | I | 31.0 ± 0.13 | 12.7 ± 0.06 | 7.8 ± 0.07 |
| 13 | I | 35.0 ± 0.15 | 12.7 ± 0.06 | 5.7 ± 0.07 |
| 16 | I | 34.6 ± 0.41 | 12.5 ± 0.22 | 5.9 ± 0.24 |
| 21 | I | 34.7 ± 0.29 | 12.5 ± 0.06 | 4.9 ± 0.05 |
| 53 | I | 35.4 ± 0.31 | 12.4 ± 0.03 | 5.7 ± 0.12 |
| 205 | I | 31.1 ± 0.12 | 12.2 ± 0.10 | 7.5 ± 0.13 |
| 210 | I | 30.8 ± 0.10 | 12.2 ± 0.09 | 7.1 ± 0.13 |
| 52 | I | 34.5 ± 0.39 | 12.1 ± 0.11 | 4.5 ± 0.07 |
| 204 | I | 30.9 ± 0.13 | 12.1 ± 0.08 | 6.9 ± 0.15 |
| 179 | I | 31.0 ± 0.10 | 11.9 ± 0.07 | 6.8 ± 0.08 |
| 209 | I | 31.7 ± 0.20 | 11.9 ± 0.12 | 6.6 ± 0.15 |
| 206 | K | 29.3 ± 0.13 | 11.3 ± 0.11 | 5.1 ± 0.09 |
| 217 | K | 29.5 ± 0.08 | 11.2 ± 0.12 | 6.0 ± 0.17 |
| 182 | K | 29.9 ± 0.18 | 11.1 ± 0.04 | 5.4 ± 0.11 |
| 214 | K | 29.6 ± 0.12 | 11.1 ± 0.15 | 4.8 ± 0.11 |
| 215 | K | 29.6 ± 0.10 | 11.1 ± 0.08 | 5.4 ± 0.08 |
| 181 | K | 29.7 ± 0.11 | 11.1 ± 0.11 | 5.9 ± 0.14 |
| 207 | I | 30.7 ± 0.10 | 11.0 ± 0.08 | 5.7 ± 0.08 |
| 178 | K | 29.7 ± 0.24 | 10.9 ± 0.12 | 5.6 ± 0.07 |
| 216 | K | 29.3 ± 0.19 | 10.8 ± 0.11 | 5.1 ± 0.16 |

[a] The samples are sorted in descending order of a* parameter. [b] The samples were classified according to a sensory analysis (Table S2). [c] The values correspond to mean ± standard deviation of a triplicate analysis. N, K and I: brown, black and intermediate chocolates, respectively

**Table S2**: Sensory analysis on the reconstituted images of the colors of the chocolates. Numbers correspond to the sample codes in Table 1

| **Samples of chocolates** | **Selection of samples** | | | | | | **Number of votes** | **Retained samples [a]** |
|---|---|---|---|---|---|---|---|---|
| | **Participant 1** | **Participant 2** | **Participant 3** | **Participant 4** | **Participant 5** | **Participant 6** | | |
| | 184 | 184 | 184 | 184 | 184 | 184 | 6 | 184 |
| | 185 | 185 | 185 | 185 | 185 | 185 | 6 | 185 |
| | 186 | 186 | 186 | 186 | 186 | 186 | 6 | 186 |
| | 15 | 15 | 15 | 15 | 15 | 15 | 6 | 15 |
| | 50 | 50 | 50 | 50 | 50 | 50 | 6 | 50 |
| | 51 | 51 | 51 | 51 | 51 | 51 | 6 | 51 |
| | 54 | 54 | 54 | 54 | 54 | 54 | 6 | 54 |
| Samples estimated as brown | 57 | 57 | 57 | 57 | 57 | 57 | 6 | 57 |
| | 183 | | 183 | 183 | | 183 | 4 | |
| | | | 17 | 17 | 17 | 17 | 4 | |
| | | | | 19 | | 19 | 2 | |
| | | | | 55 | | 55 | 2 | |
| | | | | 56 | | 56 | 2 | |
| | | | | 21 | | 21 | 2 | |
| | | | | 14 | | | 1 | |
| | | | | | | 58 | 1 | |
| | 216 | 216 | 216 | 216 | 216 | 216 | 6 | 216 |
| | 214 | 214 | 214 | 214 | 214 | 214 | 6 | 214 |
| | 217 | 217 | 217 | 217 | 217 | 217 | 6 | 217 |
| | 206 | 206 | 206 | 206 | 206 | 206 | 6 | 206 |
| | 178 | 178 | 178 | 178 | 178 | 178 | 6 | 178 |
| | 215 | 215 | 215 | 215 | 215 | 215 | 6 | 215 |
| | 181 | 181 | 181 | 181 | 181 | 181 | 6 | 181 |
| Samples estimated as black | 182 | | 182 | 182 | 182 | 182 | 5 | 182 |
| | | | | 205 | | 205 | 2 | |
| | | | | 210 | | 210 | 2 | |
| | | | | 179 | | 179 | 2 | |
| | | | | 206 | | | 1 | |
| | | | | 182 | | | 1 | |
| | | | | 181 | | | 1 | |
| | | | | 204 | | | 1 | |
| | | | | 207 | | | 1 | |

[a] The retained samples formed the groups of brown and black chocolates focused in this work and the non-retained samples formed the intermediate group

Table S4: Highly discriminating compounds for black and brown chocolates tentatively annotated by UHPLC−ESI−Q−Orbitrap MS analyses

| Type of sample | Compound code | Annotation [a] | RT (min) | Ion type | Measured *m/z* | Formula | Error (ppm) | MS/MS ion fragments (relative abundance in %) | References |
|---|---|---|---|---|---|---|---|---|---|
| Black chocolate | K1 | Protocatechuic acid [a] | 1.93 | $[M-H]^-$ | 153.0193 | C7 H6 O4 | -0.3 | 153.0193 (48), 109.0295 (100) | [7,17] |
| | K2 | Oxidized A-type procyanidin dimer | 13.84 | $[M-H]^-$ | 573.1038 | C30 H22 O12 | -0.1 | 529.1113 (7), 447.0713 (100), 325.0358 (17), 297.0408 (13), 285.0406 (66), 163.0036 (8) | [34,36] |
| | K3 | (Epi)catechin-*O*-hexoside | 6.06 | $[M-H]^-$ | 451.1239 | C21 H24 O11 | -1.5 | 289.0721 (100), 245.0820 (14), 125.0244 (10) | [7,22] |
| | K4 | A-type procyanidin dimer *O*-hexoside | 9.37 | $[M-H]^-$ | 737.1724 | C36 H34 O17 | 0.1 | 611.1396 (100), 539.0971 (36), 449.0866 (38), 407.0761 (5), 289.0714 (3) | [7,17] |
| | K5 | A-type procyanidin dimer *O*-hexoside | 12.50 | $[M-H]^-$ | 737.1724 | C36 H34 O17 | 0.1 | 611.1408 (100), 539.0984 (36), 449.0869 (37), 407.0767 (6), 289.0715 (6) | [7,17] |
| | K6 | (Epi)catechin-*O*-pentoside | 6.71 | $[M-H]^-$ | 421.1134 | C20 H22 O10 | -1.5 | 289.0718 (100), 245.0818 (19), 125.0245 (11) | |
| | K7 | (Epi)catechin-*O*-hexoside | 5.04 | $[M-H]^-$ | 451.1241 | C21 H24 O11 | -1.2 | 289.0719 (100), 245.0818 (17), 125.0244 (10) | [7,22] |
| | K8 | A-type procyanidin trimer hexoside | 10.86 | $[M-H]^-$ | 1025.2352 | C51 H46 O23 | -0.5 | 1025.2328 (100), 873.1884 (27), 735.1554 (90), 573.1249 (38), 451.1022 (18), 289.0719 (39) | [7,22] |
| | K9 | A-type procyanidin dimer *O*-pentoside | 10.37 | $[M-H]^-$ | 707.1616 | C35 H32 O16 | 0.6 | 707.1613 (100), 581.1296 (79), 539.0974 (40), 449.0867 (51), 407.0760 (3), 289.0718 (4) | [7,17] |
| | K10 | (Epi)gallocatechin-*O*-hexoside | 5.54 | $[M-H]^-$ | 467.1188 | C21 H24 O12 | -1.4 | 305.0663 (59), 287.0558 (30), 125.0244 (100) | [7] |
| | K11 | A-type procyanidin dimer *O*-pentoside | 12.82 | $[M-H]^-$ | 707.1615 | C35 H32 O16 | -0.4 | 707.1616 (100), 581.1298 (74), 539.0978 (40), 449.0867 (46), 407.0764 (7), 289.0712 (5) | [7,17] |
| | K12 | (Epi)gallocatechin-*O*-hexoside | 4.60 | $[M-H]^-$ | 467.1191 | C21 H24 O12 | -1.0 | 305.0665 (55), 287.0561 (39), 125.0244 (100) | [7] |
| | K13 | (Epi)catechin-*O*-pentoside | 7.54 | $[M-H]^-$ | 421.1133 | C20 H22 O10 | -1.8 | 289.0719 (100), 245.0816 (30), 137.0244 (25), 125.0244 (18) | |
| | K14 | A-type procyanidin dimer *O*-pentoside | 7.88 | $[M-H]^-$ | 707.1615 | C35 H32 O16 | -0.4 | 581.1298 (100), 539.0978 (40), 449.0877 (67) | [7,17] |
| | K15 | A-type procyanidin trimer pentoside | 11.87 | $[M-H]^-$ | 995.2250 | C50 H44 O22 | -0.2 | 995.2227 (100), 843.1746 (28), 705.1453 (94), 543.1132 (36), 307.0610 (96), 289.0715 (37) | [22] |
| | K16 | Unknown | 10.06 | $[M-H]^-$ | 391.1604 | C17 H28 O10 | -1.5 | 247.1187 (27), 101.0244 (30), 99.0451 (59), 57.0346 (100) | |

Table S4 : Continued

| Type of sample | Compound code | Annotation [a] | RT (min) | Ion type | Measured $m/z$ | Formula | Error (ppm) | MS/MS ion fragments (relative abundance in %) | References |
|---|---|---|---|---|---|---|---|---|---|
| Black chocolate | K17 | Unknown | 7.59 | $[M-H]^-$ | 532.2520 | C23 H39 O11 N3 | 1.6 | Non fragmented | |
| | K18 | Unknown | 9.44 | $[M-H]^-$ | 860.4117 | C34 H59 O15 N11 | -0.2 | 860.4118 (100), 486.2310 (7), 227.8464 (7), 201.0712 (10) | |
| | K19 | Unknown | 5.23 | $[M-H]^-$ | 425.1658 | C17 H30 O12 | -1.6 | 323.1347 (35), 281.1240 (92), 101.0245(51), 99.0451 (62), 57.0345 (100) | |
| | K20 | Unknown | 5.53 | $[M-H]^-$ | 542.3304 | C23 H49 O11 N3 | 1.7 | 542.3305 (100), 272.1725 (17), 242.1873 (12), 201.0710 (11) | |
| | K21 | Unknown | 6.52 | $[M-H]^-$ | 354.9989 | C4 H9 O17 N3 | 0.1 | 310.0014 (85), 280.9990 (45), 266.0123 (100), 265.0043 (44), 117.9359 (60) | |
| | K22 | Unknown | 5.37 | $[M-H]^-$ | 425.1657 | C17 H30 O12 | -1.7 | 323.1345 (32), 281.1241 (100), 101.0245 (43), 99.0451 (51), 57.0346 (85) | |
| | K23 | Methoxytyrosine | 6.52 | $[M-H]^-$ | 210.0772 | C10 H13 N O4 | -0.1 | 124.0404 (100), 94.0298 (34) | [37] |
| | K24 | Unknown | 6.73 | $[M-H]^-$ | 414.2351 | C17 H37 O10 N | 1.5 | 414.2348 (100), 242.1871 (19), 144.0778(24), 74.0247 (19) | |
| | K25 | Unknown | 6.00 | $[M-H]^-$ | 688.3534 | C30 H47 O8 N11 | -0.4 | 644.3270 (100), 184.0727 (29), 166.0621 (18), 131.0825 (16) | |
| | K26 | Unknown | 5.80 | $[M-H]^-$ | 425.1659 | C17 H30 O12 | -1.4 | 323.1346 (49), 281.1239 (83), 101.0244 (58), 99.0451 (62), 57.0345 (100) | |
| Brown chocolate | N27 | $^{13}C$ B-type procyanidin nonamer | 12.24 | $[M-2H]^{2-}$ | 1296.7869 | C135 H110 O54 | -2.2 | 863.1788 (18), 575.1196 (35), 449.0870 (7), 407.0767 (19), 289.0718 (8), 243.0300 (100) | [22,44] |
| | N28 | B-type procyanidin heptamer | 11.60 | $[M-2H]^{2-}$ | 1008.2221 | C105 H86 O42 | -0.3 | 863.1808 (59), 575.1181 (59), 449.0893 (38), 407.0773 (56), 289.0723 (59) 201.0704 (100) | [22,24,44] |
| | N29 | B-type procyanidin octamer | 11.90 | $[M-2H]^{2-}$ | 1152.2535 | C120 H98 O48 | -0.5 | 575.1187 (73), 289.0717 (39), 245.0459 (49), 243.0303 (55), 161.0248 (100) | [44] |
| | N30 | B-type procyanidin hexamer | 11.35 | $[M-2H]^{2-}$ | 864.1904 | C90 H74 O36 | -0.2 | 863.1799 (31), 575.1200 (44), 449.0866 (24), 407.0764 (55), 289.0714 (98), 161.0244 (100) | [7,22] |
| | N31 | B-type procyanidin pentamer | 10.69 | $[M-H]^-$ | 1441.3253 | C75 H62 O30 | 0.0 | 863.1766 (21), 644.1332 (16), 575.1196 (29), 407.0765 (40), 289.0712 (100), 243.0296 (37) | [7,22] |
| | N32 | B-type procyanidin hexamer | 11.01 | $[M-2H]^{2-}$ | 864.1906 | C90 H74 O36 | -0.2 | 863.1815 (24), 575.1198 (35), 451.1034 (13), 407.0765 (54), 289.0717 (96), 161.0244 (100) | [7,22] |
| | N33 | B-type procyanidin hexamer | 11.99 | $[M-2H]^{2-}$ | 864.1907 | C90 H74 O36 | 0.0 | 863.1775 (25), 575.1196 (41), 449.0868 (19), 407.0764 (47), 289.0713 (73), 161.0245 (100) | [7,22] |
| | N34 | B-type procyanidin tetramer | 9.98 | $[M-H]^-$ | 1153.2617 | C60 H50 O24 | -0.2 | 983.2024 (14), 865.1980 (17), 739.1669 (10), 575.1189 (27), 289.0715 (15), 125.0245 (100) | [7,43] |

Table S4 : Continued

| Type of sample | Compound code | Annotation [a] | RT (min) | Ion type | Measured $m/z$ | Formula | Error (ppm) | MS/MS ion fragments (relative abundance in %) | References |
|---|---|---|---|---|---|---|---|---|---|
| | N35 | Unknown | 5.73 | $[M-H]^-$ | 890.3382 | C34 H53 O19 N9 | -0.3 | 890.3361 (100), 872.3269 (88), 854.3199 (48), 369.1787 (45), 201.0706 (43), 145.0619 (52) | |
| | N36 | $^{13}$C B-type procyanidin pentamer | 10.40 | $[M-2H]^{2-}$ | 720.6605 | C74 $^{13}$C H62 O30 | -0.2 | 289.0712 (66), 287.0573 (49), 243.0298 (93), 217.0505 (48), 201.0709 (100), 151.0406 (48) | [7,17,22] |
| | N37 | B-type procyanidin tetramer | 10.25 | $[M-2H]^{2-}$ | 576.1271 | C60 H50 O24 | -0.4 | 575.1195 (37), 407.0763 (22), 287.0557 (29), 243.0299 (30), 177.0193 (14), 125.0244 (100) | [7,22] |
| | N38 | $^{13}$C B-type procyanidin hexamer | 8.55 | $[M-2H]^{2-}$ | 864.6924 | C89 $^{13}$C H72 O36 | 0.1 | 407.0756 (66), 289.0721 (89), 287.0567 (71), 245.0459 (60), 243.0295 (100) | [7,22] |
| | N39 | B-type procyanidin pentamer | 13.54 | $[M-2H]^{2-}$ | 720.1590 | C75 H62 O30 | 0.0 | 863.1824 (10), 577.1347 (21), 449.0871 (14), 407.0769 (80), 289.0716 (51), 161.0245 (100) | [7,22] |
| | N40 | $^{13}$C B-type procyanidin pentamer | 11.22 | $[M-2H]^{2-}$ | 720.6605 | C74 $^{13}$C H62 O30 | -0.2 | Non fragmented | |
| | N41 | Unknown | 6.09 | $[M-H]^-$ | 872.3279 | C34 H51 O18 N9 | 0.0 | 872.3260 (100), 854.3148 (19), 369.1768 (19), 223.1089 (10), 145.0619 (18), 127.0514 (12) | |
| Brown chocolate | N42 | B-type procyanidin tetramer | 7.74 | $[M-H]^-$ | 1153.2617 | C60 H50 O24 | -0.2 | 983.2030 (15), 865.1980 (15), 575.1191 (29), 407.0764 (21), 289.0713 (15), 125.0244 (100) | [7,43] |
| | N43 | B-type procyanidin tetramer | 13.96 | $[M-H]^-$ | 1153.2617 | C60 H50 O24 | -0.2 | 983.2037 (18), 865.1976 (28), 575.1195 (22), 407.0763 (35), 289.0715 (21), 125.0244 (100) | [7,43] |
| | N44 | Syringic acid hexoside | 4.55 | $[M-H]^-$ | 359.0977 | C15 H20 O10 | -1.8 | 197.0456 (100), 182.0221 (37), 153.0557 (23) | [7] |
| | N45 | Dehydrodicatechin B [b] | 7.32 | $[M-H]^-$ | 577.1350 | C30 H26 O12 | -0.2 | 577.1342 (100), 559.1252 (4), 439.1028 (12), 425.0865 (8), 393.0966 (7) | [39] |
| | N46 | B-type procyanidin pentamer | 8.97 | $[M-2H]^{2-}$ | 720.1590 | C75 H62 O30 | 0.0 | 577.1340 (18), 575.1205 (21), 407.0769 (40), 289.0717 (100), 175.0401 (25) | [7,22] |
| | N47 | B-type procyanidin trimer | 9.10 | $[M-H]^-$ | 865.1983 | C45 H38 O18 | -0.3 | 695.1397 (27), 577.1349 (33), 451.1028 (12), 407.0766 (35), 289.0719 (29), 125.0244 (100) | [7,17] |
| | N48 | A-type procyanidin dimer (A2) [a] | 12.30 | $[M-H]^-$ | 575.1195 | C30 H24 O12 | 1.0 | 575.1183 (100), 449.0868 (34), 407.0768 (11), 285.0406 (53), 125.0243 (42) | [7,17,22] |
| | N49 | B-type procyanidin trimer | 8.40 | $[M-H]^-$ | 865.1984 | C45 H38 O18 | -0.1 | 695.1390 (43), 577.1342 (57), 425.0865 (24), 287.0567 (35), 125.0244 (100) | [7,17] |
| | N50 | B-type procyanidin pentamer | 7.76 | $[M-2H]^{2-}$ | 720.1591 | C75 H62 O30 | 0.1 | 863.1812 (12), 577.1357 (13), 575.1181 (28), 449.0872 (20), 407.0767 (45), 289.0715 (100) | [7,22] |

Table S4 : Continued

| Type of sample | Compound code | Annotation [a] | RT (min) | Ion type | Measured $m/z$ | Formula | Error (ppm) | MS/MS ion fragments (relative abundance in %) | References |
|---|---|---|---|---|---|---|---|---|---|
| | N51 | Unknown | 7.58 | $[M-H]^-$ | 449.1083 | C21 H22 O11 | -1.4 | Non fragmented | |
| | N52 | B-type procyanidin tetramer | 7.48 | $[M-H]^-$ | 1153.2616 | C60 H50 O24 | -0.2 | 865.1979 (18), 575.1203 (29), 407.0766 (19), 289.0718 (12), 125.0244 (100) | [7,43] |
| | N53 | Dehydrodicatechin B [b] | 7.78 | $[M-H]^-$ | 577.1350 | C30 H26 O12 | -0.2 | 577.1340 (100), 559.1267 (2), 533.1457 (5), 439.1029 (18), 425.0868 (8), 393.0959 (8) | [39] |
| | N54 | A-type procyanidin dimer | 13.57 | $[M-H]^-$ | 575.1195 | C30 H24 O12 | 1.0 | 449.0870 (69), 423.0721 (40), 285.0405 (98), 125.0243 (100) | [7,17,22] |
| | N55 | B-type procyanidin trimer *C*-hexoside | 8.15 | $[M-H]^-$ | 1027.2509 | C51 H48 O23 | -0.5 | 907.2056 (12), 577.1348 (41), 425.0878 (18), 407.0766 (24), 287.0774 (6), 125.0245 (100) | [22] |
| | N56 | B-type procyanidin trimer | 12.79 | $[M-H]^-$ | 865.1984 | C45 H38 O18 | -0.1 | 695.1396 (50), 577.1348 (42), 451.1027 (32), 425.0873 (31), 407.0766 (100), 289.0718 (51) | [7,17] |
| | N57 | Unknown | 8.93 | $[M-H]^-$ | 378.1664 | C18 H25 O6 N3 | -1.7 | 360.1558 (64), 343.1293 (100), 299.1402 (15), 164.0717 (40), 152.0718 (29) | |
| | N58 | B-type procyanidin tetramer | 10.91 | $[M-H]^-$ | 1153.2616 | C60 H50 O24 | -0.2 | 865.1996 (10), 577.1347 (27), 407.0765 (20), 289.0715 (12), 125.0244 (100) | [7,43] |
| Brown chocolate | N59 | B-type procyanidin trimer | 6.94 | $[M-H]^-$ | 865.1981 | C45 H38 O18 | -0.5 | 695.1399 (25), 577.1350 (27), 451.1028 (10), 407.0766 (36), 289.0719 (26), 125.0244 (100) | [7,17] |
| | N60 | B-type procyanidin tetramer | 6.64 | $[M-H]^-$ | 1153.2615 | C60 H50 O24 | -0.4 | 865.1969 (16), 577.1357 (23), 407.0770 (25), 287.0559 (30), 125.0244 (100) | [7,43] |
| | N61 | B-type procyanidin trimer | 12.98 | $[M-H]^-$ | 865.1985 | C45 H38 O18 | -0.1 | 695.1385 (26), 577.1343 (24), 425.0868 (11), 407.0765 (35), 289.0720 (30), 125.0243 (100) | [7,17] |
| | N62 | B-type procyanidin trimer | 10.41 | $[M-H]^-$ | 865.1984 | C45 H38 O18 | -0.2 | 695.1394 (16), 577.1348 (29), 425.0875 (18), 407.0766 (34), 289.0719 (22), 125.0244 (100) | [7,17] |
| | N63 | Feruloyl aspartic acid | 7.62 | $[M-H]^-$ | 308.0776 | C14 H15 N O7 | 0.0 | 308.0774 (100), 246.0771 (9), 193.0507 (25), 149.0608 (36), 134.0373 (42) | [7,19] |
| | N64 | Unknown | 2.03 | $[M-H]^-$ | 479.1400 | C19 H28 O14 | 0.4 | 433.1349 (76), 293.0879 (100), 233.0665 (27), 125.0242 (34), 101.0244 (12), 71.0138 (52) | |
| | N65 | B-type procyanidin trimer | 6.46 | $[M-H]^-$ | 865.1981 | C45 H38 O18 | -0.5 | 695.1399 (25), 577.1349 (28), 451.1029 (11), 407.0766 (37), 289.0721 (30), 125.0244 (100) | [7,17] |
| | N66 | Unknown | 6.96 | $[M-H]^-$ | 591.1144 | C30 H24 O13 | -0.1 | Non fragmented | |

Table S4 : Continued

| Type of sample | Compound code | Annotation [a] | RT (min) | Ion type | Measured *m/z* | Formula | Error (ppm) | MS/MS ion fragments (relative abundance in %) | References |
|---|---|---|---|---|---|---|---|---|---|
| | N67 | Unknown | 7.10 | $[M-H]^-$ | 403.1617 | C18 H28 O10 | 1.7 | 403.1620 (100), 257.0930 (24), 145.0618 (40), 127.0513 (47), 109.0407 (20) | |
| | N68 | Unknown | 2.24 | $[M-H]^-$ | 293.1144 | C14 H18 N2 O5 | 0.2 | Non fragmented | |
| | N69 | Unknown | 5.78 | $[M-H]^-$ | 501.1470 | C18 H30 O16 | 1.7 | 179.0573 (26), 151.0625 (78), 101.0244 (26), 99.0452 (67), 59.0138 (13), 57.0345 (100) | |
| | N70 | Unknown | 4.94 | $[M-H]^-$ | 323.1348 | C13 H24 O9 | 0.0 | 323.1349 (100), 201.0709 (6), 119.0350 (15), 101.0244 (26), 89.0244 (41), 59.0138 (79) | |
| | N71 | B-type procyanidin dimer *C*-hexoside | 6.53 | $[M-H]^-$ | 739.1881 | C36 H36 O17 | 0.1 | 649.1564 (10), 619.1460 (50), 449.0842 (12), 329.0664 (38), 289.0716 (48), 167.0350 (100) | [7,22] |
| | N72 | Phenylalanylphenylalanine | 11.42 | $[M-H]^-$ | 311.1401 | C18 H20 N2 O3 | 0.0 | 311.1402 (100), 175.0878 (16), 174.9561 (8), 164.0718 (11), 147.0452 (10), 91.0553 (24), | [45] |
| | N73 | Unknown | 2.26 | $[M-H]^-$ | 433.1346 | C18 H26 O12 | -1.4 | 293.0879 (65), 233.0666 (43), 125.0245 (44), 71.0138 (82), 59.0138 (100) | |
| | N74 | Unknown | 7.49 | $[M-H]^-$ | 582.1252 | C28 H25 O13 N | -0.2 | 582.1244 (100), 466.1148 (12), 449.0877 (26), 433.9951 (19), 423.1065 (31), 243.0302 (24) | |
| | N75 | (Epi)catechin derivative | 8.46 | $[M-H]^-$ | 561.1399 | C30 H26 O11 | -0.6 | 435.1082 (17), 407.0756 (13), 289.0716 (100), 245.0820 (14), 125.0244 (31) | |
| | N76 | (Epi)catechin hexoside-sulfate | 5.16 | $[M-H]^-$ | 531.0809 | C21 H24 O14 S | -0.9 | 531.0806 (100), 289.0717 (40), 245.0820 (9), 137.0245 (16), 125.0242 (5) | |

[a] Annotation confirmed by authentic standard. [b] Compound having a $\beta$-interflavanic configuration [39]. RT: retention time

**Figure S1**: Loading plot of principal component analysis from the UHPLC−HRMS features of the black and brown chocolates. Blue dots correspond to discriminating features for black (bottom group) and brown (top group) chocolates obtained from the differential analysis in Figure 4. The corresponding score plot of PCA is shown in Figure 3.

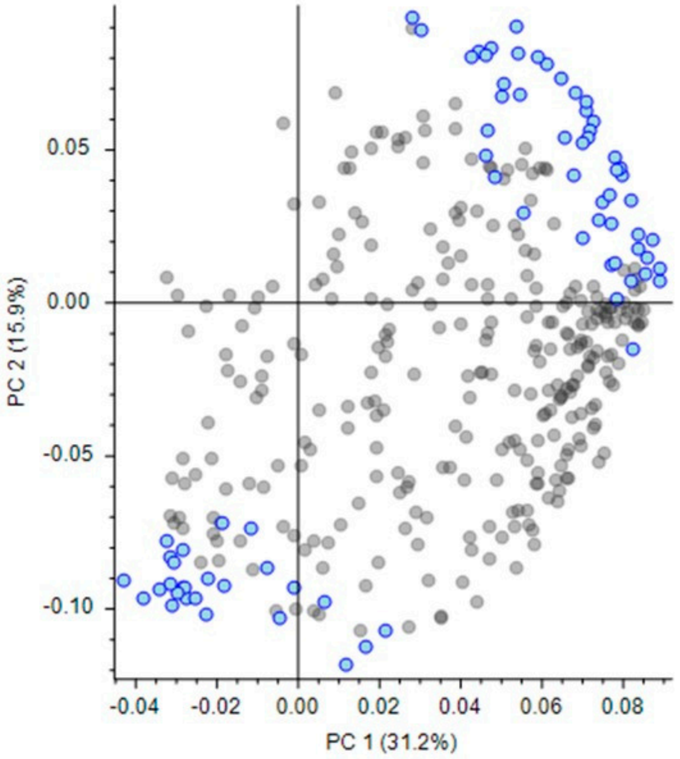

Supplement: Supplementary file 1 [file metabolites-13-00667-s001.zip › metabolites-2372229-supplementary.pdf]
